# Lactate to hemoglobin ratio predicts short and long term mortality in critically ill patients with Gastrointestinal bleeding

**DOI:** 10.1038/s41598-025-27176-6

**Published:** 2025-12-05

**Authors:** Qinglin Wu, Fulan Cen, Ying Xie, Xianjia Ning, Jinghua Wang, Zhenghao Lin, Jia Huang

**Affiliations:** 1 Department of Intensive Care Unit, Shenzhen Third People’s Hospital, Shenzhen, 518112 Guangdong Province China; 2 Graduate School of Public Administration, Seoul National University, Seoul, South Korea; 3 Center of Clinical Epidemiology, Shenzhen Third People’s Hospital, Shenzhen, Guangdong China; 4 Department of Intensive Care Unit, Shenzhen Third People’s Hospital, 29 Bulan Road, Longgang District, Shenzhen, 518112 Guangdong Province China

**Keywords:** Lactate-to-hemoglobin ratio, Gastrointestinal bleeding, Mortality, ICU, MIMIC-IV, Diseases, Gastroenterology, Health care

## Abstract

This study investigates the lactate-to-hemoglobin ratio (LHR) as a novel biomarker for predicting short-term and long-term mortality in critically ill patients with gastrointestinal bleeding (GIB). Data from 1387 patients in the MIMIC - IV database were analyzed. Results showed that LHR was significantly associated with 7-day, 28-day and 365-day mortality. It had moderate discriminative ability in predicting mortality, with area under the curve (AUC) values ranging from 0.637 to 0.694. Compared with traditional markers and scoring systems, LHR has certain advantages. However, the study has limitations such as a retrospective design and single - center data. In conclusion, LHR shows promise as a prognostic tool for GIB patients, but further research is needed.

**Supplementary Information:**

The online version contains supplementary material available at 10.1038/s41598-025-27176-6.

## Introduction

Gastrointestinal bleeding (GIB) is a severe clinical event with substantial global health implications. It leads to significant morbidity and mortality, accounting for a large number of hospital admissions and healthcare costs annually. In the United States alone, GIB results in over 500,000 hospitalizations each year, making it one of the leading causes of gastrointestinal-related hospital readmission and mortality^1,2^. In the intensive care unit (ICU) setting, the incidence of GIB reaches approximately 1.3%, with mortality rates as high as 47%, emphasizing the critical need for early detection and intervention^3,4^.

Recent advancements have led to the development of various scoring systems designed to predict the prognosis of GIB patients. Tools such as the Rockall score, Glasgow-Blatchford score (GBS), and AIMS65 score have proven useful for risk stratification, especially in cases involving upper gastrointestinal bleeding (UGIB)^5,6^. However, while effective, these systems often rely on complex calculations or parameters that are difficult to implement rapidly in urgent care environments or resource-limited settings^7^. Furthermore, they may not fully account for the diverse clinical scenarios present in ICU patients, where the bleeding risk can be exacerbated by multiple comorbidities^8^.

Despite these advances, there remains a gap in the development of simple, easily accessible, and cost-effective prognostic markers for GIB^9^. Currently used indicators, such as lactate and hemoglobin levels, offer promise but are often limited by factors such as patient condition and the influence of concurrent diseases like sepsis or liver dysfunction^10^. While elevated lactate levels are associated with poor outcomes in GIB patients, their prognostic utility is compromised by these confounding factors^11,12^. Similarly, hemoglobin levels provide a rapid estimate of blood loss but may fail to reflect the severity of bleeding in the acute phase^13^. Consequently, there is an unmet need for a combined marker that is both practical and predictive in ICU settings, where timely risk assessment is paramount.

The present study aims to address this gap by evaluating the serum lactate to hemoglobin ratio (LHR) as a potential prognostic marker in patients with GIB. We hypothesize that the LHR can offer a more accurate prediction of both short- and long-term all-cause mortality in critically ill patients with both UGIB and lower gastrointestinal bleeding (LGIB) admitted to the ICU. This study seeks to establish the efficacy of LHR as a cost-effective and reliable tool for predicting mortality, thereby improving patient stratification and outcomes in critical care settings.

## Materials and methods

### Study design

This study adopted a retrospective research design with the aim of exploring the lactate - to - hemoglobin ratio as a novel biomarker for predicting short - and long - term mortality in critically ill patients with gastrointestinal bleeding. We intended to clarify the relationship between the LHR and the mortality outcomes of these patients.

### Data sources

The *Medical Information Mart for Intensive Care* database [MIMIC-IV (version 2.2)], an extensive and publicly accessible resource developed by the *Massachusetts Institute of Technology’s Laboratory of Computational Physiology*, provided all data for this study^14^. Data extraction was authorized for the author, Qinglin Wu (Author ID: 13,442,112). The MIMIC-IV database is approved by the *Institutional Review Board (IRB) of Beth Israel Deaconess Medical Center* (IRB Protocol #2001P001699) with a waiver of informed consent. In line with national legislation and institutional requirements, written informed consent was not necessary for this study.

### Participants

**Fig. 1:**
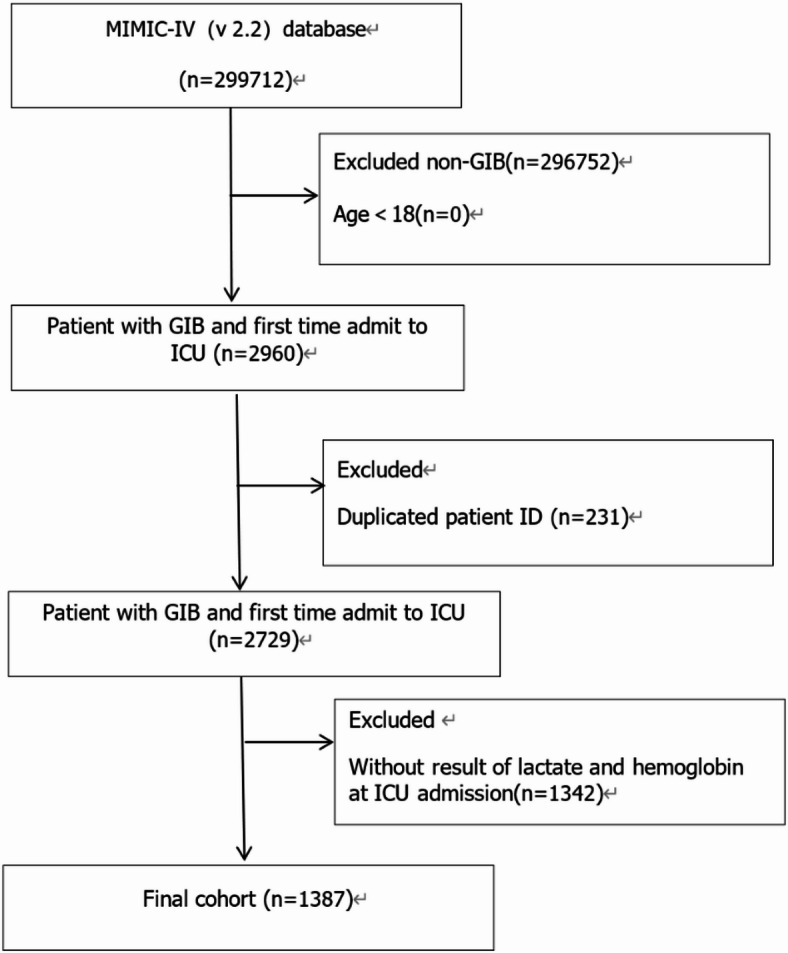
Flowchart for participants from the MIMIC-IV.

The current study included patients with both UGIB and LGIB who were over the age of 18 at the time of their initial ICU hospitalization. The following were the GIB diagnostic criteria used in the present research the major associated *International Classification of Diseases* (ICD) codes contained: ICD-9:578.0,578.1,578.9, ICD-10: K92.s0, K92.1, K92 and complete ICD diagnostic codes were available in the supplementary materials(Supplementary material S1). The following groups were omitted from this set of data:^1^ people under 18 years of age;^2^ people lacking result of lactate and hemoglobin at ICU admission within 24 h;^3^ Duplicated patient ID. Finally, 1387 patients were included in the study. The specific selection process is shown in (Fig. 1). No priori sample-size calculation was performed for LHR. The present study is hypothesis-generating and estimation-oriented, with emphasis on precision (95% CI) rather than formal hypothesis testing. We calculated the Events Per Variable (EPV) for each end point. The EPV for the 7-day mortality endpoint was 17.1 (number of events = 171, number of variables = 10), for the 28-day mortality endpoint it was 26.9 (number of events = 404, number of variables = 15), and for the 365-day mortality endpoint it was 42.3 (number of events = 677, number of variables = 16). All values exceeded the commonly used threshold of 10.

### Data extraction

The LHR was selected as the primary study variable. Serum lactate and hemoglobin levels were assessed immediately after admission to minimize treatment-related interference. All variables were extracted from the MIMIC-IV database utilizing Structured Query Language (SQL) with PostgreSQL. Collect demographics about the patient [age, gender, body mass index (BMI) and ethnicity], vital signs [heart rate (HR), mean blood pressure (MBP), systolic blood pressure (SBP), respiratory rate (RR), temperature], acute kidney injure (AKI), comorbidities [renal disease, malignant cancer, liver disease, myocardial infarction (MI), congestive heart failure (CHF), cerebrovascular disease, chronic pulmonary disease, peptic ulcer, diabetes], clinical treatments [(ventilator, continuous renal replacement therapy (CRRT)], laboratory tests [white blood cell (WBC), neutrophils, red blood cell (RBC), platelet, hemoglobin, hematocrit, creatinine, blood urea nitrogen (BUN), albumin (ALB), total bilirubin (TB), glucose, international normalized ratio (INR), prothrombin time (PT), partial thromboplastin time (PTT), lactate and the lactate to hemoglobin ratio was calculated (LHR)], various scores[*systemic inflammatory response* syndrome (SIRS), *acute physiology score III* (APSIII), *sequential organ failure assessment score* (SOFA). A comprehensive list of the extracted variables is provided in Table 1. The outcomes of this research were 7-day, 28-day and 365-day mortality. We also conducted a sample size assessment.

### Statistical analysis

We used normality tests and Q-Q plots to determine whether the distribution of variables followed a normal distribution (Supplementary material S2). Continuous variables were presented as mean ± standard deviation (SD) for normally distributed data or median (P25, P75) for non-normally distributed data. Categorical variables were expressed as frequencies and percentages. After data extraction, we conducted statistics and analysis on the missing data conditions of baseline variables. The number and proportion of missing data for each variable are as follows: ALB 664 (47.87%), Neutrophils 628 (45.28%), SOFA 387 (27.90%), TB 308 (22.21%), PTT 66 (4.76%), INR 60 (4.33%), PT 60 (4.33%), Temperature 48 (3.46%), SBP 12 (0.87%), SIRS 9 (0.65%), Glucose 3 (0.22%), HR 3 (0.22%), MBP 3 (0.22%), RR 3 (0.22%). Given the high proportion of missing data in ALB, Neutrophils, SOFA, and TB, these variables were excluded from further analysis to ensure the accuracy of statistical analysis. For the remaining variables that did not follow a normal distribution based on normality tests, we applied median imputation.

Separate univariable analyses, collinearity diagnostics, and multivariable analyses were performed for LHR treated as a continuous variable and categorized as a grouped variable.

Univariate analyses were initially conducted to evaluate the association between the LHR and different clinical endpoints. We conducted collinearity diagnostics for variables with a univariate P-value < 0.05 before including them in the multivariate binary logistic regression analysis. We assessed collinearity using the Variance Inflation Factor (VIF > 5) and tolerance(<0.2), and combined these assessments with the clinical significance of the variables for a comprehensive evaluation (Supplementary material S3). For 7-day mortality, hemoglobin (VIF = 19.335), hematocrit (VIF = 21.97), INR (VIF = 8.475), and PT (VIF = 8.621) were excluded. For 28-day mortality, INR and PT were excluded as their VIF values were greater than 5. Variables identified as collinear were excluded, while the remaining variables were included in the multivariate binary logistic regression analysis. OR and 95% confidence intervals (CI) were calculated to estimate the strength of associations between LHR quartiles and mortality outcomes. When LHR is treated as a grouping variable in the analysis, the values of LHR were divided into four quartiles (Q1-Q4) based on LHR levels, with Q3 defined as the 50th to 75th percentile. Using Q1 (the lowest quartile) as the reference group, all analyses calculated odds ratios (ORs) for Q2, Q3, and Q4 against this reference. The analysis focused on determining the predictive value of LHR for 7-day, 28-day and 365-day mortality. Receiver Operating Characteristic (ROC) curve analysis was performed to evaluate the predictive accuracy of LHR. The area under the curve (AUC) was calculated to assess the discriminative ability of these markers. The cutoff for the ROC (Receiver Operating Characteristic) curve was determined using the best Youden index. All statistical analyses were performed using SPSS Statistics (Version 26, IBM, Chicago, IL, USA), and R (version 4.2.0). A two-tailed P-value of less than 0.05 was considered statistically significant.

## Results

### Baseline demographic and clinical characteristics

The baseline characteristics of patients with both UGIB and LGIB in the MIMIC-IV database (2008–2019) consisting of 1,387 patients with gastrointestinal bleeding, were included in this study (Fig. 1) and detailed in Table 1. 582 (42.0%) were women, and 805 were men (58.0%). The mean age was 65.96 years (women: 68.05, men: 64.44). Patients were categorized into three age groups: ≤64 years (44.3%), 65–79 years (33.5%), and ≥ 80 years (22.2%). Ethnic distribution were White (66.7%), followed by Unknown (18.7%), Black (8.4%), Asian (3.2%), and others (3.0%).Overall BMI: 28.90 kg/m² (women normal BMI: 35.4%, men: 27.4%). Vital signs at admission contained a median HR of 94.0 beats/min (IQR: 80.0–109.0), SBP of 118.0 mmHg (IQR: 101.0–137.0), and RR of 20.0 breaths/min (IQR: 16.0–24.0). The median temperature was 36.67℃ (IQR: 36.39–37.06). Severity scores had a median value of 3.0 for SIRS (IQR: 2.0–4.0), 64.0 for APSIII (IQR: 47.0–87.0), and 3.0 for SOFA (IQR: 2.0–5.0). Comorbidities included high rates of AKI (80.0%) and CHF (32.9%). Laboratory results included median lactate levels of 2.00 mmol/L (IQR: 1.30–3.10), hemoglobin of 9.60 g/dL (IQR: 8.10–11.40), and creatinine of 1.30 mg/dL (IQR: 0.80–2.20). The LHR was divided into quartiles: Q1 (0.1068, IQR: 0.0086–0.1220), Q2 (0.1667, IQR: 0.1500–0.1845), Q3 (0.2532, IQR: 0.2283–0.2821), and Q4 (0.4848, IQR: 0.3852–0.7387). Different points of deaths and mortality: 7-day 12.3% (171/1387), 28-day 29.1% (404/1387), 365-day 48.8% (677/1387).

**Table 1:** Characteristics of participants.

Characteristics	Men	Women	Total
Case, n (%)	805(58)	582(42)	1387
**Age**,** means ± SD**	64.44 ± 15.95	68.05 ± 16.38	65.96 ± 16.22
**Age groups**,** n (%)**			
≤ 64years	395(49.1)	219(37.6)	614(44.3)
65-79years	258(32.0)	207(35.6)	465(33.5)
≥ 80years	152(18.9)	156(26.8)	308(22.2)
***Ethnicities n(%)***			
White	532(66.1)	393(67.5)	925(66.7)
Black	65(8.1)	52(8.9)	117(8.4)
Asian	28(3.5)	16(2.7)	44(3.2)
Others	22(2.7)	20(3.4)	42(3.0)
Unknown*	158(19.6)	101(17.4)	259(18.7)
***BMI***,*** means ± SD***	29.19 ± 8.87	28.51 ± 7.83	28.90 ± 8.45
***BMI groups***			
Normal (18.5 ≤ BMI<24)	138(27.4)	130(35.4)	268(30.8)
Overweight (24 ≤ BMI<28)	183(36.3)	101(27.5)	284(32.6)
Obesity (28 ≤ BMI)	183(36.3)	136(37.1)	319(36.6)
***Vital Signs***,*** median(25%***,***75%)***			
HR (beat/min)	94.0(80.0,109.0)	94.0(80.0,108.0)	94.0(80.0,109.0)
MBP(mmHg)	80.0(68.0,93.8)	77.0(66.0,91.0)	78.0(67.0,93.0)
SBP (mmHg)	119.0(102.0,137.0)	117.0(101.0,134.5)	118.0(101.0,137.0)
RR (time/min)	20.0(17.0,24.0)	20.0(16.0,24.0)	20.0(16.0,24.0)
Temperature(℃)	36.72(36.39,37.06)	36.67(36.39,37.00)	36.67(36.39,37.06)
***Severity score***,*** median(25%***,***75%)***			
SIRS	3.0(2.0,4.0)	3.0(2.0,3.0)	3.0(2.0,4.0)
APSIII	64.0(48.0,88.0)	64.0(47.0,87.0)	64.0(47.0,87.0)
SOFA	3.0(2.0,6.0)	3.0(1.0,5.0)	3.0(2.0,5.0)
AKI	652(81.0)	458(78.7)	1110(80.0)
***Comorbidities n(%)***			
Renal disease	251(31.2)	125(21.5)	376(27.1)
Malignant cancer	141(17.5)	83(14.3)	224(16.1)
Liver disease	294(36.5)	174(29.9)	468(33.7)
Myocardial infarction	176(21.9)	113(19.4)	289(20.8)
Congestive heart failure	264(32.8)	193(33.2)	457(32.9)
Cerebrovascular disease	91(11.3)	73(12.5)	164(11.8)
Chronic pulmonary disease	193(24.0)	184(31.6)	377(27.2)
Peptic ulcer	188(23.4)	119(20.4)	307(22.1)
Diabetes	261(32.4)	177(30.4)	438(31.6)
Ventilator	690(85.7)	495(85.1)	1185(85.4)
CRRT	114(14.2)	85(14.6)	199(14.3)
***Laboratory results***,*** median(25%***,***75%)***			
White blood cell(×10^3^/uL)	12.00(8.10,17.60)	12.30(8.50,17.40)	12.20(8.30,17.50)
Neutrophils(×10^3^/uL)	9.91(6.00,15.01)	11.13(6.59,15.82)	10.38(6.28,15.35)
Red blood cell (×10^6^/uL)	3.20(2.68,3.85)	3.17 (2.69,3.72)	3.18(2.68,3.78)
Platelet (×10^3^/uL)	164.00(104.00,246.00)	175.50(103.75,256.50)	168.00(104.00,249.00)
Hemoglobin(g/dL)	9.70(8.20,11.60)	9.50(8.00,11.10)	9.60(8.10,11.40)
Hematocrit(%)	29.60(24.75,35.2)	29.05(24.68,34.00)	29.30(24.70,34.80)
Creatinine (mg/dL)	1.40(0.90,2.40)	1.10(0.70,1.90)	1.30(0.80,2.20)
Blood urea nitrogen (mg/dL)	32.00(20.00,57.00)	27.50(17.00,49.25)	30.00(18.00,54.00)
Albumin(g/dL)	2.80(2.40,3.30)	2.90(2.45,3.30)	2.90(2.40,3.30)
TB (mg/dL)	1.20(0.60,3.50)	1.10(0.50,3.30)	1.10(0.50,3.40)
Glucose (mg/dL)	136.00(110.00,182.00)	136.00(108.00,177.00)	136.00(109.00,179.00)
INR	1.40(1.20,1.80)	1.40(1.20,1.90)	1.40(1.20,1.80)
PT (second)	15.30(13.13,19.70)	15.20(13.20,20.40)	15.30(13.20,19.90)
PTT(second)	33.20(28.00,44.40)	32.75(27.88,43.23)	33.00(28.00,43.90)
Lactate(mmol/L)	2.00(1.30,3.10)	2.00(1.30,3.20)	2.00(1.30,3.10)
LHR*	0.1977(0.1343,0.3333)	0.2169(0.1397,0.3389)	0.2051(0.1379,0.3333)
***LHR groups***,*** median(25%***,***75%)***			
Q1	0.1062(0.090,0.1220)	0.1081(0.0866,0.1237)	0.1068(0.0086,0.1220)
Q2	0.1688(0.1517,0.1855)	0.1644(0.1476,0.1836)	0.1667(0.1500,0.1845)
Q3	0.2500(0.2254,0.2766)	0.2615(0.2308,0.2870)	0.2532(0.2283,0.2821)
Q4	0.4920 (0.3925,0.7693)	0.4632(0.3810,0.7029)	0.4848(0.3852,0.7387)

**Notes**: SD: Standard deviation. BMI: Body mass index. HR: Heart rate. MBP: Mean blood pressure. SBP: Systolic blood pressure. RR: Respiratory rate. SIRS: Systemic inflammatory response syndrome. APSIII: Acute physiology score III. SOFA: Sequential organ failure assessment score. AKI: Acute kidney injury. CRRT: Continuous renal replacement therapy. WBC: White blood cell. RBC: Red blood cell. INR: International normalized ratio. PT: Prothrombin time. PTT: Partial thromboplastin time. LHR: Lactate - to - hemoglobin ratio. The group classified as “unknown” in Ethnicities groups is composed of entries labeled as “UNKNOWN”, “PATIENT DECLINED TO ANSWER”, and “UNABLE TO OBTAIN” in the original dataset. *Statistical analyses treated lactate-to-hemoglobin ratio as a continuous variable.

### Univariate binary logistic regression of LHR in patients with GIB

Table 2 showed the results of the univariate Binary Logistic Regression of clinical characteristics related to mortality in patients with gastrointestinal bleeding, focusing on 7-day, 28-day, and 365-day mortality. Age was not significantly associated with 7-day or 28-day mortality, but showed positive association with 365-day mortality (OR = 1.020, 95% CI: 1.013–1.027, *P* < 0.001), with risk increasing with age. Ethnicity had no significant impact on mortality. Heart rate had non-significant association with mortality. MBP and SBP were inversely related to 28-day and 365-day mortality. Respiratory rate was positively associated with mortality at all end points (e.g., 7-day: OR = 1.036, 95% CI: 1.011–1.062, *P* = 0.005), while temperature was negatively associated. SIRS, APSIII, and AKI were strongly associated with increased mortality at all time points (e.g., AKI for 7-day mortality: OR = 5.089, 95% CI: 2.566–10.092, *P* < 0.001). Among comorbidities, renal disease was associated with 365-day mortality (OR = 1.272, 95% CI: 1.003–1.613, *P* = 0.047), malignant cancer with 28-day and 365-day mortality, and liver disease with all-cause mortality at different time points. The utilization of ventilation and CRRT had no statistically significant impact on mortality outcomes. Multiple hematologic and biochemical markers, including WBC, RBC, hemoglobin levels, serum creatinine, and lactate concentrations, exhibited significant associations with mortality across distinct temporal endpoints (*P* < 0.05). For instance, lactate was positively associated with mortality at all time points (7-day: OR = 1.263, 95% CI: 1.200–1.330, *P* < 0.001).

Statistical analysis of LHR as a continuous variable: OR 4.302 (95% CI 2.901–6.378, *p* < 0.001) for 7-day; OR 4.992 (95% CI 3.273–7.613, *p* < 0.001) for 28-day; OR 5.269 (95% CI 3.232–8.589, *p* < 0.001) for 365-day mortality. Quartile analysis of LHR (vs. Q1 reference): Q4 had the most prominent increase, with 7-day mortality OR = 4.917 (95% CI: 2.939–8.227, *P* < 0.001), 28-day mortality OR = 4.565 (95% CI: 3.179–6.557, *P* < 0.001), and 365-day mortality OR = 3.519 (95% CI: 2.580–4.806, *P* < 0.001),.

Variables with a P - value < 0.05 under different mortality outcomes were included in subsequent collinearity tests for further screening (Supplementary material S3). We compared two backbone multivariate models to avoid mathematical coupling and assess the incremental value. Model 1 consisted of the clinical covariates plus LHR, while Model 2 consisted of the same clinical covariates plus its components. The difference in the Area Under the Curve (ΔAUC) between these models was evaluated using DeLong’s test. As the ΔAUC was negligible (< 0.01) across all endpoints, indicating no meaningful improvement in discriminatory power, we retained the more parsimonious Model 1 (including LHR but excluding lactate) for the final analysis(Supplementary material S4).

**Table 2:** Results of the univariate binary logistic regression of clinical characteristics.

Characteristics	7d mortality		28d mortality		365d mortality	
	OR(95%CI)	*P*	OR(95%CI)	*P*	OR(95%CI)	*P*
*Age*	0.995(0.986,1.005)	0.346	1.005(0.998,1.012)	0.175	1.020(1.013,1.027)	**< 0.001**
***Ethnicities***		0.453		0.501		0.484
White	**Reference**		**Reference**		**Reference**	
Black	1.300(0.748,2.258)	0.352	1.073(0.707,1.628)	0.742	0.916(0.623,1.346)	0.655
Asian	1.966(0.920,4.202)	0.081	1.126(0.588,2.157)	0.720	1.197(0.652,2.198)	0.561
Others	1.033(0.397,2.686)	0.947	0.483(0.212,1.100)	0.083	0.748(0.401,1.398)	0.363
Unknown*	1.116(0.736,1.694)	0.605	1.002(0.741,1.356)	0.988	0.809(0.614,1.068)	0.134
***Vital Signs***						
HR (beat/min)	1.007(1.000,1.015)	0.067	1.005(0.999,1.011)	0.074	1.000(0.995,1.005)	0.968
MBP(mmHg)	0.997(0.989,1.005)	0.418	0.993(0.987,0.999)	**0.019**	0.993(0.988,0.998)	**0.008**
SBP (mmHg)	0.994(0.987,1.000)	0.053	0.992(0.988,0.997)	**0.001**	0.992(0.988,0.997)	**< 0.001**
RR(time/min)	1.036(1.011,1.062)	**0.005**	1.037(1.018,1.057)	**< 0.001**	1.035(1.017,1.053)	**< 0.001**
Temperature(℃)	0.664(0.575,0.768)	**< 0.001**	0.693(0.612,0.785)	**< 0.001**	0.728(0.645,0.822)	**< 0.001**
***Severity score***						
SIRS	2.171(1.734,2.717)	**< 0.001**	1.571(1.357,1.818)	**< 0.001**	1.207(1.066,1.367)	**0.003**
APSIII	1.035(1.029,1.041)	**< 0.001**	1.039(1.034,1.045)	**< 0.001**	1.029(1.025,1.034)	**< 0.001**
AKI	5.089(2.566,10.092)	**< 0.001**	4.762(3.142,7.219)	**< 0.001**	2.927(2.199,3.898)	**< 0.001**
***Comorbidities*** n(%)						
Renal disease	0.714(0.486,1.050)	0.087	1.045(0.806,1.354)	0.742	1.272(1.003,1.613)	**0.047**
Malignant cancer	1.149(0.768,1.720)	0.500	1.827(1.374,2.429)	**< 0.001**	3.390(2.509,4.581)	**< 0.001**
Liver disease	1.636(1.182,2.265)	**0.003**	1.773(1.395,2.253)	**< 0.001**	1.565(1.251,1.958)	**< 0.001**
MI	0.975(0.656,1.449)	0.899	1.083(0.817,1.437)	0.578	1.053(0.812,1.364)	0.698
CHF	0.782(0.567,1.077)	0.132	0.956(0.757,1.207)	0.703	1.155(0.934,1.428)	0.184
Cerebrovascular disease	0.689(0.395,1.202)	0.189	0.910(0.632,1.310)	0.612	0.999(0.721,1.384)	0.993
Chronic pulmonary disease	1.123(0.789,1.599)	0.518	1.344(1.042,1.734)	**0.023**	1.263(0.996,1.600)	0.054
Peptic ulcer	1.085(0.743,1.586)	0.672	1.307(0.996,1.715)	0.054	1.333(1.034,1.719)	0.270
Diabetes	0.774(0.540,1.107)	0.161	0.800(0.620,1.031)	0.085	0.926(0.738,1.161)	0.503
Ventilator	0.853(0.552,1.318)	0.474	1.256(0.893,1.767)	0.190	1.280(0.948,1.729)	0.107
CRRT	0.918(0.575,1.466)	0.721	1.059(0.763,1.470)	0.731	1.121(0.830,1.514)	0.456
***Laboratory results***						
WBC(×10^3^/uL)	1.007(0.996,1.017)	0.211	1.017(1.005,1.030)	**0.004**	1.014(1.003,1.026)	**0.017**
RBC (×10^6^/uL)	1.346(1.121,1.616)	**0.001**	1.032(0.899,1.184)	0.655	0.908(0.801,1.029)	0.131
Platelet (×10^3^/uL)	0.998(0.997,1.000)	**0.015**	0.999(0.998,1.000)	0.244	0.999(0.998,1.000)	0.141
Hemoglobin(g/dL)	1.160(1.089,1.234)	**< 0.001**	1.045(0.997,1.095)	0.067	0.973(0.932,1.015)	0.206
Hematocrit(%)	1.053(1.032,1.075)	**< 0.001**	1.018(1.002,1.034)	**0.024**	0.998(0.984,1.012)	0.764
Creatinine (mg/dL)	1.110(1.032,1.195)	**0.005**	1.087(1.023,1.156)	**0.007**	1.128(1.058,1.201)	**< 0.001**
BUN (mg/dL)	1.006(1.001,1.010)	**0.013**	1.007(1.004,1.011)	**< 0.001**	1.005(1.005,1.012)	**< 0.001**
Glucose (mg/dL)	1.001(1.000,1.003)	0.151	1.000(0.999,1.002)	0.637	0.999(0.998,1.000)	0.112
INR	1.248(1.108,1.407)	**< 0.001**	1.389(1.229,1.570)	**< 0.001**	1.315(1.158,1.493)	**< 0.001**
PT (second)	1.022(1.010,1.033)	**< 0.001**	1.031(1.019,1.043)	**< 0.001**	1.025(1.013,1.037)	**< 0.001**
PTT(second)	1.009(1.004,1.014)	**< 0.001**	1.012(1.008,1.016)	**< 0.001**	1.007(1.003,1.012)	**0.001**
Lactate(mmol/L)	1.263(1.200,1.330)	**< 0.001**	1.266(1.201,1.335)	**< 0.001**	1.238(1.170,1.311)	**< 0.001**
LHR*	4.302(2.901,6.378)	**< 0.001**	4.992(3.273,7.613)	**< 0.001**	5.269(3.232,8.589)	**< 0.001**
***LHR groups***		**< 0.001**		**< 0.001**		**< 0.001**
Q1	**Reference**		**Reference**		**Reference**	
Q2	1.103(0.591,2.061)	0.758	1.893(1.292,2.773)	**0.001**	1.664(1.222,2.265)	**0.001**
Q3	2.580(1.494,4.456)	**0.001**	2.555(1.759,3.710)	**< 0.001**	2.318(1.702,3.156)	**< 0.001**
Q4	4.917(2.939,8.227)	**< 0.001**	4.565(3.179,6.557)	**< 0.001**	3.519(2.580,4.806)	**< 0.001**

**Notes**: OR: Odds ratio. 95% CI: 95% confidence interval. HR: Heart rate (beats/min). MBP: Mean blood pressure (mmHg). SBP: Systolic blood pressure (mmHg). RR: Respiratory rate (breaths/min). SIRS: Systemic inflammatory response syndrome. APSIII: Acute Physiology Score III. AKI: Acute kidney injury. MI: Myocardial infarction. CHF: Congestive heart failure. WBC: White blood cell count (×10³/µL). RBC: Red blood cell count (×10⁶/µL). INR: International normalized ratio. PT: Prothrombin time (seconds). PTT: Partial thromboplastin time (seconds). LHR: Lactate-to-hemoglobin ratio (mmol/L ÷ g/dL). BUN: Blood urea nitrogen (mg/dL). *Statistical analyses treated lactate-to-hemoglobin ratio as a continuous variable.

### Multivariate binary logistic regression of LHR in patients with GIB

After adjusting for potential confounders through multivariate binary logistic regression, LHR remained significantly associated with mortality across all time points when analyzed as a continuous variable (Table 3). For 7-day mortality, the adjusted odds ratio was 2.445 (95% CI: 1.559–3.948, *P* < 0.001). When analyzed by quartiles, the LHR groups showed a significant overall association with 7-day mortality (*P* = 0.045), with the Q3 group exhibiting a significantly elevated risk compared to Q1 (OR = 1.880, 95% CI: 1.039–3.403, *P* = 0.037). Other important predictors for 7-day mortality included RBC, SIRS score, APSIII, and lactate level.

For 28-day mortality, LHR as a continuous variable remained a significant predictor (OR = 2.042, 95% CI: 1.304–3.312, *P* = 0.003). In the quartile-based analysis, both Q2 (OR = 1.631, 95% CI: 1.066–2.496, *P* = 0.024) and Q3 (OR = 1.738, 95% CI: 1.126–2.682, *P* = 0.013) were associated with increased mortality risk compared to Q1, while Q4 showed a trend toward significance (OR = 1.734, 95% CI: 0.997–3.013, *P* = 0.051). Additional significant predictors for 28-day mortality included APSIII, AKI, malignant cancer, hematocrit, and PTT.

In the analysis of 365-day mortality, LHR as a continuous variable was again significantly associated with mortality (OR = 2.074, 95% CI: 1.285–3.522, *P* = 0.005). Among LHR quartiles, only Q3 demonstrated a statistically significant increase in risk compared to Q1 (OR = 1.550, 95% CI: 1.075–2.235, *P* = 0.019). Age, APSIII, malignant cancer, and AKI were also independently associated with 365-day mortality.

**Table 3:** Multivariate binary logistic regression of between patients and mortality.

	7-day mortality		28-day mortality		365-day mortality	
Characteristics	Adjusted-OR (95%CI)	*P*	Adjusted-OR (95%CI)	*P*	Adjusted-OR (95%CI)	*P*
LHR*	2.445 (1.559, 3.948)	< 0.001	2.042 (1.304, 3.312)	0.003	2.074 (1.285, 3.522)	0.005
*LHR groups*		0.045		0.066		0.132
**Q1**	**Reference**		**Reference**		**Reference**	
**Q2**	0.921(0.477,1.780)	0.807	**1.631(1.066**,**2.496)**	**0.024**	1.303(0.921,1.844)	0.135
**Q3**	**1.880(1.039**,**3.403)**	**0.037**	**1.738(1.126**,**2.682)**	**0.013**	**1.550(1.075**,**2.235)**	**0.019**
**Q4**	1.678(0.840,3.352)	0.143	**1.734(0.997**,**3.013)**	**0.051**	1.471(0.900,2.401)	0.124
**Age**					**1.033(0.998**,**1.042)**	**< 0.001**
MBP			1.002 (0.992, 1.012)	0.675	1.007 (0.999, 1.016)	0.100
SBP			0.999 (0.992, 1.007)	0.880	0.996 (0.989, 1.003)	0.229
RR	0.999(0.970, 1.028)	0.939	1.002 (0.979, 1.025)	0.866	1.015 (0.995, 1.037)	0.151
T	0.898 (0.769, 1.053)	0.179	**0.862 (0.749**,** 0.991)**	**0.037**	0.847 (0.736, 0.971)	0.018
SIRS	**1.429(1.111**,** 1.850)**	**0.006**	1.068 (0.879, 1.274)	0.462	0.928 (0.795, 1.083)	0.344
APSIII	**1.026 (1.019**,** 1.033)**	**< 0.001**	**1.031 (1.025**,** 1.038)**	**< 0.001**	**1.024 (1.019**,** 1.030)**	**< 0.001**
AKI			**2.077 (1.330**,** 3.349)**	**0.002**	**1.557 (1.116**,** 2.184)**	**0.010**
Malignant cancer			**2.195 (1.565**,** 3.078)**	**< 0.001**	**3.714 (2.660**,** 5.236)**	**< 0.001**
Liver disease	1.144 (0.771, 1.687)	0.500	1.181 (0.877, 1.586)	0.271	**1.760 (1.307**,** 2.378)**	**< 0.001**
Chronic pulmonary disease			**1.399 (1.039**,** 1.881)**	**0.026**		
Renal disease					1.095 (0.807, 1.486)	0.559
WBC					1.005 (0.994, 1.018)	0.408
RBC	**1.602 (1.300**,** 1.975)**	**< 0.001**				
Hematocrit			**1.027 (1.008**,** 1.047)**	**0.006**		
Creatinine	0.968 (0.856, 1.082)	0.589	**0.889 (0.802**,** 0.980)**	**0.021**	1.011 (0.923, 1.106)	0.806
BUN	1.005 (0.998, 1.011)	0.167	**1.008 (1.002**,** 1.013)**	**0.005**	1.005 (0.998, 1.011)	0.167
PTT	1.003 (0.996, 1.009)	0.415	**1.007 (1.002**,** 1.012)**	**0.006**	1.003 (0.998, 1.008)	0.287

**Notes**: Adjusted-OR: Adjusted odds ratio. 95% CI: 95% confidence interval. LHR: Lactate-to-hemoglobin ratio (mmol/L ÷ g/dL). MBP: Mean blood pressure (mmHg). SBP: Systolic blood pressure (mmHg). RR: Respiratory rate (breaths/min). T: Temperature (°C). SIRS: Systemic inflammatory response syndrome. APSIII: Acute Physiology Score III. AKI: Acute kidney injury. WBC: White blood cell count (×10³/µL). RBC: Red blood cell count (×10⁶/µL). Hematocrit: Percentage of blood volume occupied by red blood cells. Creatinine: Serum creatinine (mg/dL). BUN: Blood urea nitrogen (mg/dL). PTT: Partial thromboplastin time (seconds).*Statistical analyses treated lactate-to-hemoglobin ratio as a continuous variable.

### ROC curve analysis

**Fig. 2:**
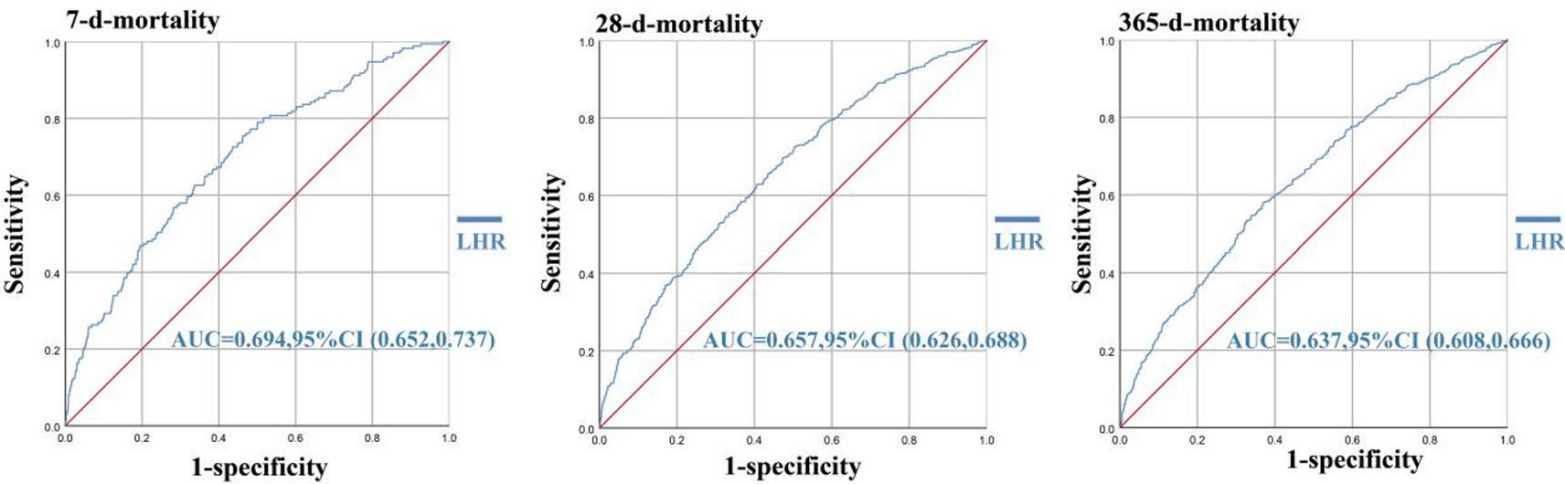
ROC curves for predicting all-cause mortality in patients with different outcomes. ROC curves for 7-day, 28-day, and 365-day all-cause mortality. Curves are labeled with corresponding mortality endpoints and area under the curve (AUC) with 95% confidence intervals (CI).

ROC curves were generated for various mortality endpoints, including, 7-day, 28-day and 365-day mortality (Fig. 2). The LHR demonstrated moderate discriminative ability across all mortality endpoints. For 7-day mortality, the AUC was 0.694 (95% CI: 0.652–0.737; *P* < 0.001) For 28-day mortality, the AUC decreased to 0.657 (95% CI: 0.626–0.688; *P* < 0.001). For 365-day mortality, the AUC decreased to 0.637 (95% CI: 0.608–0.666; *P* < 0.001). Different cutoff points determined by the Youden index were tested for their sensitivity and specificity in predicting mortality (Supplementary material S5).

## Discussion

To our knowledge, this is the first study to evaluate the association between the LHR and mortality of patients with GIB. Our findings suggested that the LHR may be a significant independent predictor of both short- and long-term mortality in critically ill patients with GIB admitted to the ICU. The results indicated that LHR appears to be a predictor of mortality, with higher LHR values being associated with an increased probability of death at the 7-, 28-, and 365-day endpoints. In particular, the LHR Q3 group showed a higher likelihood of 28-day death than the Q1 group in the multivariate binary logistic regression analysis. In the ROC curve analysis, LHR showed moderate discriminative ability for all mortality endpoints, with the highest AUC value of 0.694 for 7-day mortality. These findings suggest that LHR can effectively stratify risk not only in the acute phase but also in predicting long-term outcomes, which is crucial for informing prognosis and tailoring therapeutic strategies in ICU settings. Moreover, the association between elevated LHR and adverse outcomes remained significant even after adjusting for multiple confounding factors. This suggests that LHR may have potential as an independent biomarker for risk stratification. While quartile analysis demonstrated a dose-dependent risk increase in Q2 and Q3, Q4 did not reach statistical significance in multivariate models of the 28-day mortality. Extreme values in Q4 might reflect rare clinical scenarios (e.g., acute liver failure, massive hemorrhage), introducing variability that dilutes effect estimates. And variables such as APSIII, AKI, and malignant cancer (strong mortality predictors) were included in the model, potentially attenuating the independent effect of Q4. Patients in Q4 may overrepresent those with severe comorbidities, leading to effect modification rather than true loss of association. This highlights the need for larger cohorts to validate optimal cutoff points and explore potential ceiling effects in LHR-mortality relationships.

Compared to traditional indicators such as lactate or hemoglobin alone, LHR offers several distinct advantages. Lactate, although widely recognized as a marker of tissue hypoperfusion and cellular distress, is susceptible to fluctuations due to factors like sepsis or metabolic disturbances, which can compromise its predictive accuracy^15,16^. Similarly, hemoglobin levels, while reflecting the degree of blood loss, may not capture the acute changes in oxygen delivery status that accompany severe bleeding or hemorrhagic shock^17,18^. By combining these two parameters, the LHR provides a more comprehensive assessment that integrates both the severity of blood loss and the resulting systemic hypoxia, thus offering a more stable and reliable prognostic tool^19^. This is especially relevant in critically ill patients, where dynamic changes in physiological status require biomarkers that can rapidly respond to shifts in clinical conditions. As our study shows, LHR outperforms these individual markers, providing a nuanced evaluation of patient risk, which could lead to earlier identification of high-risk patients and more timely intervention.

The observed dose-response relationship between LHR and mortality risk of GIB supports its use as a reliable marker for risk stratification better than other marker like Red Blood Cell Distribution Width (RDW) or LAR^20,21^. This finding is in line with previous studies demonstrating that lactate-based biomarkers are useful for mortality prediction in other acute settings, such as sepsis and trauma, where they reflect both the severity of the initial insult and the adequacy of the patient’s physiological response^22,23^.Our study extends this application to GIB, indicating that LHR may serve as a universal marker for predicting adverse outcomes across a range of critical illnesses. Furthermore, the utility of LHR in long-term mortality prediction suggests that it can be incorporated into discharge planning and long-term follow-up strategies, providing clinicians with valuable information for guiding post-ICU management and improving overall patient outcomes.

Compared to conventional risk assessment tools such as the Rockall and AIMS65 scores, A Canadian study indicated that for the outcome of death, the AUC was higher at 0.73 (95% CI: 0.69–0.78)^24^. In a study involving 251 patients with GIB, the AUC for predicting in-hospital mortality using the AIMS65 score was 0.74. In this study, the AUC of LHR was slightly lower than that of Rockall and AIMS65 scores^25^. However, LHR offers a simpler, more efficient alternative that can be readily integrated into routine clinical practice^26^. Traditional scoring systems like the Glasgow-Blatchford score (GBS) and Rockall score are primarily validated for use in UGIB and require multiple clinical and endoscopic parameters to be accurately calculated^27^. These tools, while effective in specialized settings, may be impractical in urgent care environments or resource-limited settings due to their complexity and reliance on parameters that are not always available at the time of presentation^28^. In contrast, the LHR can be rapidly calculated from standard laboratory tests that are routinely performed upon ICU admission, making it a feasible option for early risk stratification in patients with GIB. This streamlined approach could facilitate more timely decision-making and reduce the burden on healthcare resources, particularly in settings where access to specialized scoring systems is limited.

The ROC curve analysis provides further evidence of the utility of LHR, with AUC values ranging from 0.637 to 0.694, indicating moderate discriminatory power across different mortality endpoints. The highest AUC was observed for 7-day mortality (AUC = 0.694), suggesting that LHR is most effective for short-term risk prediction when early intervention is crucial^29^. However, its moderate AUC values imply that LHR should ideally be used in conjunction with other clinical markers or scoring systems to enhance its predictive accuracy, particularly for long-term outcomes.

Overall, our findings suggest that LHR may be a potentially useful prognostic marker for ICU patients with GIB. It offers a relatively practical and accessible approach for early risk stratification. Future studies should focus on validating these findings in larger, multicenter cohorts and developing standardized LHR thresholds for diverse clinical settings, ensuring that its potential can be fully realized across various patient populations.

## Limitations

This study has several limitations. First, the retrospective design limits the ability to establish causality between LHR and mortality, and unmeasured confounders may influence the observed associations, potentially affecting the validity of the findings. Second, the use of data from a single-center database (MIMIC-IV) may limit the generalizability of the results to other ICU settings and populations, making it less applicable to broader or more diverse patient cohorts. Third, a significant limitation arising from the data source is our inability to report the proportion of patients with UGIB versus LGIB within the overall GIB cohort. This is primarily due to the inherent limitations of the ICD coding system used in the MIMIC-IV database. Some relevant ICD codes (e.g., K92.2 “unspecified gastrointestinal hemorrhage”) lack anatomical specificity to reliably distinguish UGIB from LGIB. Furthermore, the structured data extraction approach employed did not capture critical clinical information required for accurate differentiation (such as endoscopic or radiological reports detailing the bleeding source). This inability to stratify the cohort by bleeding site is a substantial limitation, as UGIB and LGIB differ significantly in etiology, management strategies, and prognosis. Consequently, the prognostic performance of LHR might vary across these distinct entities, and readers should exercise caution when interpreting the findings, recognizing that they represent an amalgamation of both UGIB and LGIB patients. Additionally, due to the limitations of the database, we were unable to further stratify the etiological types of GIB. Such stratification may have facilitated a more in-depth investigation of the role of LHR in different GIB-related diseases. Furthermore, despite adjustments for multiple confounders, residual confounding from unmeasured variables, such as additional treatments during ICU admission, may still be present, potentially influencing the results. The event count for the 7-day endpoint was slightly fewer for, which may overestimate the statistical potential. To address these limitations, future research should focus on conducting prospective, multicenter studies with larger and more diverse populations, ensuring adequate long-term follow-up and incorporating a wider range of clinical variables to reduce potential confounding.

## Conclusion

The considerable prognostic utility of LHR in predicting both short- and long-term mortality for patients with GIB was highlighted in this cohort. The results of this study only affect the studied cohort. Increased mortality is closely associated with higher LHR values, providing a potential non-invasive risk stratification tool that can assist in early identification of high-risk patients for prompt therapies to promote survival and lower morbidity. Nevertheless, this study’s retrospective design, single-center data, and incapacity to stratify GIB etiological categories are major drawbacks. Future studies should focus on further validating the use of LHR in diverse patient populations and clinical settings, as well as exploring its integration into existing risk assessment models for GIB.

## Supplementary Information

Below is the link to the electronic supplementary material.

Supplementary Material 1

Supplementary Material 2

Supplementary Material 3

Supplementary Material 4

Supplementary Material 5

## Data Availability

The MIMIC-IV (version 2.2) database, an extensive and publicly accessible resource developed by the Massachusetts Institute of Technology’s Laboratory of Computational Physiology, provided all data for this study. Data extraction was authorized for the author, Qinglin Wu (Author ID: 13,442,112). The data will be made available on reasonable request. Readers can contact for the data request through：Author：Qinglin Wu, Email：wuqinglin5700@163.com.

## Data availability

The MIMIC-IV (version 2.2) database, an extensive and publicly accessible resource developed by the Massachusetts Institute of Technology’s Laboratory of Computational Physiology, provided all data for this study. Data extraction was authorized for the author, Qinglin Wu (Author ID: 13,442,112). The data will be made available on reasonable request. Readers can contact for the data request through: Author༚Qinglin Wu, Email༚wuqinglin5700@163.com.
