# Supplementary material for: Lactate to hemoglobin ratio predicts short and long term mortality in critically ill patients with Gastrointestinal bleeding
Source: Sci Rep. 2025 Dec 5;15:43216. doi: 10.1038/s41598-025-27176-6 (PMC12680706; doi:10.1038/s41598-025-27176-6)
Supplement: Supplementary file 1 — Supplementary Material 1 [file 41598_2025_27176_MOESM1_ESM.docx]

**Supplementary material S1- GIB disease codes in SQL**

'K51311','53101','53460','K266','53141','K256','K5721','53200','53501','K5781','K9401','53551','53160','53100','K2901','K272','53341','K5741','K625','53161','K250','K2971','K9411','53301','K226','53221','K51211','5781','53361','K2931','53421','53531','5789','53320','K50111','K51011','K9431','53521','K2991','53400','K2941','K2961','53561','K6381','K51911','K5521','K2211','K3182','K284','53300','53260','53220','K270','K51411','53360','K2981','K282','53082','K286','K2951','53201','53511','53240','53401','53461','5307','53440','K51511','K260','53261','K51811','K262','53784','53120','53340','53441','K276','53021','K264','K252','53321','K50911','53241','K50011','K2921','53121','K31811','K254','5780','53420','53140','K9421','53783','K280','K274','K50811','53541','K5701','K92','K920','K921','K922'
