# Supplementary material for: Lactate to hemoglobin ratio predicts short and long term mortality in critically ill patients with Gastrointestinal bleeding
Source: Sci Rep. 2025 Dec 5;15:43216. doi: 10.1038/s41598-025-27176-6 (PMC12680706; doi:10.1038/s41598-025-27176-6)
Supplement: Supplementary file 2 — Supplementary Material 2 [file 41598_2025_27176_MOESM2_ESM.docx]

**Supplementary material S2- Normality test result of laboratory results**

| ***Laboratory results*** | **Kolmogorov Smirnov test^a^** | **df** | **P value** | **Shapiro-Wilk test** | **df** | **P value** |
| --- | --- | --- | --- | --- | --- | --- |
| **WBC** | 0.120 | 455.000 | ＜0.001 | 0.733 | 455.000 | ＜0.001 |
| **Neutrophils** | 0.115 | 455.000 | ＜0.001 | 0.751 | 455.000 | ＜0.001 |
| **RBC** | 0.062 | 455.000 | ＜0.001 | 0.980 | 455.000 | ＜0.001 |
| **Platelet** | 0.099 | 455.000 | ＜0.001 | 0.918 | 455.000 | ＜0.001 |
| **hemoglobin** | 0.055 | 455.000 | 0.002 | 0.986 | 455.000 | ＜0.001 |
| **hematocrit** | 0.052 | 455.000 | 0.006 | 0.986 | 455.000 | ＜0.001 |
| **Creatinine** | 0.194 | 455.000 | ＜0.001 | 0.715 | 455.000 | ＜0.001 |
| **BUN** | 0.154 | 455.000 | ＜0.001 | 0.820 | 455.000 | ＜0.001 |
| **ALB** | 0.055 | 455.000 | 0.002 | 0.991 | 455.000 | 0.007 |
| **TB** | 0.286 | 455.000 | ＜0.001 | 0.572 | 455.000 | ＜0.001 |
| **Glucose** | 0.172 | 455.000 | ＜0.001 | 0.710 | 455.000 | ＜0.001 |
| **INR** | 0.235 | 455.000 | ＜0.001 | 0.587 | 455.000 | ＜0.001 |
| **PT** | 0.232 | 455.000 | ＜0.001 | 0.553 | 455.000 | ＜0.001 |
| **PTT** | 0.231 | 455.000 | ＜0.001 | 0.653 | 455.000 | ＜0.001 |
| **Lacate** | 0.229 | 455.000 | ＜0.001 | 0.627 | 455.000 | ＜0.001 |
| **LHR** | 0.241 | 455.000 | ＜0.001 | 0.552 | 455.000 | ＜0.001 |

**a.Riley's Significance Correction**
