# Supplementary material for: Lactate to hemoglobin ratio predicts short and long term mortality in critically ill patients with Gastrointestinal bleeding
Source: Sci Rep. 2025 Dec 5;15:43216. doi: 10.1038/s41598-025-27176-6 (PMC12680706; doi:10.1038/s41598-025-27176-6)
Supplement: Supplementary file 3 — Supplementary Material 3 [file 41598_2025_27176_MOESM3_ESM.docx]

**Supplementary material S3- Results of collinearity test**

**Multicollinearity Analysis Result Using LHR as a Grouping Variable of different endpoint**

| **7-day mortality** |  |  | **28-day mortality** |  |  | **365-day mortality** |  |  |
| --- | --- | --- | --- | --- | --- | --- | --- | --- |
|  | **Tolerance** | **VIF** |  | **Tolerance** | **VIF** |  | **Tolerance** | **VIF** |
| **RR** | 0.9 | 1.111 | **MBP** | 0.465 | 2.15 | **age** | 0.733 | 1.365 |
| **T** | 0.888 | 1.126 | **SBP** | 0.461 | 2.171 | **MBP** | 0.464 | 2.155 |
| **SIRS** | 0.834 | 1.199 | **RR** | 0.905 | 1.105 | **SBP** | 0.458 | 2.184 |
| **APSIII** | 0.644 | 1.553 | **T** | 0.879 | 1.137 | **RR** | 0.909 | 1.1 |
| **Liver disease** | 0.773 | 1.294 | **SIRS** | 0.834 | 1.199 | **T** | 0.875 | 1.143 |
| **RBC** | **0.107** | **9.324** | **APSIII** | 0.589 | 1.699 | **SIRS** | 0.825 | 1.212 |
| **Platelet** | 0.831 | 1.203 | **AKI** | 0.857 | 1.167 | **APSIII** | 0.589 | 1.698 |
| **Hemoglobin** | **0.052** | **19.335** | **Malignant cancer** | 0.945 | 1.058 | **AKI** | 0.839 | 1.192 |
| **Hematocrit** | **0.046** | **21.97** | **Liver disease** | 0.839 | 1.191 | **Rena disease** | 0.753 | 1.328 |
| **Creatinine** | 0.607 | 1.648 | **Chronic pulmonary disease** | 0.974 | 1.027 | **Malignant cancer** | 0.949 | 1.054 |
| **BUN** | 0.573 | 1.745 | **WBC** | 0.948 | 1.055 | **Liver disease** | 0.74 | 1.351 |
| **INR** | **0.118** | **8.475** | **Hematocrit** | 0.83 | 1.204 | **WBC** | 0.947 | 1.056 |
| **PT** | **0.116** | **8.621** | **Creatinine** | 0.601 | 1.665 | **Creatinine** | 0.552 | 1.811 |
| **PTT** | 0.909 | 1.101 | **BUN** | 0.57 | 1.756 | **BUN** | 0.524 | 1.908 |
| **Lactate** | 0.52 | 1.922 | **INR** | **0.118** | **8.451** | **INR** | **0.118** | **8.454** |
| **LHR groups** | 0.534 | 1.873 | **PT** | **0.116** | **8.616** | **PT** | **0.116** | **8.61** |
|  |  |  | **PTT** | 0.9 | 1.111 | **PTT** | 0.903 | 1.108 |
|  |  |  | **Lactate** | 0.521 | 1.92 | **Lactate** | 0.537 | 1.861 |
|  |  |  | **LHR groups** | 0.537 | 1.861 | **LHR groups** | 0.577 | 1.734 |

**Re-examining Multicollinearity After Removing Associated Variables**

| **7-day mortality** |  |  | **28-day mortality** |  |  | **365-day mortality** |  |  |
| --- | --- | --- | --- | --- | --- | --- | --- | --- |
|  | **Tolerance** | **VIF** |  | **Tolerance** | **VIF** |  | **Tolerance** | **VIF** |
| **RR** | 0.903 | 1.108 | **MBP** | 0.465 | 2.15 | **Age** | 0.734 | 1.363 |
| **T** | 0.891 | 1.122 | **SBP** | 0.463 | 2.162 | **MBP** | 0.464 | 2.155 |
| **SIRS** | 0.841 | 1.189 | **RR** | 0.905 | 1.105 | **SBP** | 0.459 | 2.176 |
| **APSIII** | 0.651 | 1.535 | **T** | 0.88 | 1.137 | **RR** | 0.909 | 1.1 |
| **Liver disease** | 0.808 | 1.237 | **SIRS** | 0.838 | 1.194 | **T** | 0.875 | 1.143 |
| **RBC** | 0.855 | 1.17 | **APSIII** | 0.592 | 1.688 | **SIRS** | 0.829 | 1.206 |
| **Platelet** | 0.874 | 1.144 | **AKI** | 0.857 | 1.167 | **APSIII** | 0.592 | 1.689 |
| **Creatinine** | 0.614 | 1.629 | **Malignant cancer** | 0.949 | 1.054 | **AKI** | 0.839 | 1.192 |
| **BUN** | 0.59 | 1.696 | **Liver disease** | 0.852 | 1.173 | **Rena disease** | 0.753 | 1.328 |
| **PTT** | 0.943 | 1.061 | **Chronic pulmonary disease** | 0.974 | 1.027 | **Malignant cancer** | 0.952 | 1.051 |
| **Lactate** | 0.53 | 1.887 | **WBC** | 0.948 | 1.055 | **Liver disease** | 0.749 | 1.335 |
| **LHR groups** | 0.541 | 1.847 | **Hematocrit** | 0.837 | 1.195 | **WBC** | 0.947 | 1.056 |
|  |  |  | **Creatinine** | 0.604 | 1.656 | **Creatinine** | 0.554 | 1.804 |
|  |  |  | **BUN** | 0.581 | 1.72 | **BUN** | 0.536 | 1.864 |
|  |  |  | **PTT** | 0.928 | 1.077 | **PTT** | 0.93 | 1.075 |
|  |  |  | **Lactate** | 0.524 | 1.909 | **Lactate** | 0.539 | 1.855 |
|  |  |  | **LHR groups** | 0.54 | 1.853 | **LHR groups** | 0.581 | 1.721 |

**Supplementary material S3- Results of collinearity test**

**Multicollinearity Analysis Result Using LHR as a Continuous variable of different endpoint**

| **7-day mortality** |  |  | **28-day mortality** |  |  | **365-day mortality** |  |  |
| --- | --- | --- | --- | --- | --- | --- | --- | --- |
|  | **Tolerance** | **VIF** |  | **Tolerance** | **VIF** |  | **Tolerance** | **VIF** |
| **RR** | 0.904 | 1.106 | **MBP** | 0.474 | 2.108 | **age** | 0.720 | 1.389 |
| **T** | 0.883 | 1.133 | **SBP** | 0.468 | 2.136 | **MBP** | 0.470 | 2.129 |
| **SIRS** | 0.822 | 1.217 | **RR** | 0.910 | 1.099 | **SBP** | 0.466 | 2.148 |
| **APSIII** | 0.640 | 1.563 | **T** | 0.869 | 1.151 | **RR** | 0.910 | 1.099 |
| **Liver disease** | 0.778 | 1.286 | **SIRS** | 0.823 | 1.215 | **T** | 0.864 | 1.157 |
| **RBC** | **0.106** | **9.430** | **APSIII** | 0.588 | 1.700 | **SIRS** | 0.809 | 1.236 |
| **Platelet** | 0.835 | 1.197 | **AKI** | 0.855 | 1.170 | **APSIII** | 0.589 | 1.696 |
| **Hemoglobin** | **0.052** | **19.149** | **Malignant cancer** | 0.946 | 1.057 | **AKI** | 0.845 | 1.184 |
| **Hematocrit** | **0.047** | **21.459** | **Liver disease** | 0.846 | 1.182 | **Rena disease** | 0.758 | 1.319 |
| **Creatinine** | 0.596 | 1.677 | **Chronic pulmonary disease** | 0.973 | 1.028 | **Malignant cancer** | 0.945 | 1.058 |
| **BUN** | 0.567 | 1.763 | **WBC** | 0.949 | 1.054 | **Liver disease** | 0.734 | 1.363 |
| **INR** | **0.119** | **8.375** | **Hematocrit** | 0.517 | 1.936 | **WBC** | 0.945 | 1.058 |
| **PT** | **0.117** | **8.539** | **Creatinine** | 0.592 | 1.690 | **Creatinine** | 0.545 | 1.835 |
| **PTT** | 0.899 | 1.112 | **BUN** | 0.565 | 1.771 | **BUN** | 0.517 | 1.934 |
| **Lactate** | 0.069 | **14.531** | **INR** | **0.120** | **8.337** | **INR** | **0.120** | **8.339** |
| **LHR** | 0.069 | **14.545** | **PT** | **0.117** | **8.518** | **PT** | **0.117** | **8.517** |
|  |  |  | **PTT** | 0.893 | 1.120 | **PTT** | 0.897 | 1.115 |
|  |  |  | **Lactate** | **0.071** | **14.005** | **Lactate** | **0.118** | **8.494** |
|  |  |  | **LHR** | **0.073** | **13.743** | **LHR** | **0.126** | **7.917** |

**Re-examining Multicollinearity After Removing Associated Variables**

| **7-day mortality** |  |  | **28-day mortality** |  |  | **365-day mortality** |  |  |
| --- | --- | --- | --- | --- | --- | --- | --- | --- |
|  | **Tolerance** | **VIF** |  | **Tolerance** | **VIF** |  | **Tolerance** | **VIF** |
| **RR** | 0.928 | 1.101 | **MBP** | 0.474 | 2.108 | **Age** | 0.734 | 1.363 |
| **T** | 0.890 | 1.123 | **SBP** | 0.469 | 2.133 | **MBP** | 0.464 | 2.155 |
| **SIRS** | 0.828 | 1.208 | **RR** | 0.912 | 1.096 | **SBP** | 0.459 | 2.176 |
| **APSIII** | 0.661 | 1.514 | **T** | 0.872 | 1.147 | **RR** | 0.909 | 1.1 |
| **Liver disease** | 0.809 | 1.235 | **SIRS** | 0.827 | 1.209 | **T** | 0.875 | 1.143 |
| **RBC** | 0.853 | 1.173 | **APSIII** | 0.608 | 1.646 | **SIRS** | 0.829 | 1.206 |
| **Platelet** | 0.876 | 1.142 | **AKI** | 0.858 | 1.166 | **APSIII** | 0.592 | 1.689 |
| **Creatinine** | 0.603 | 1.660 | **Malignant cancer** | 0.947 | 1.056 | **AKI** | 0.839 | 1.192 |
| **BUN** | 0.59 | 1.694 | **Liver disease** | 0.851 | 1.175 | **Rena disease** | 0.753 | 1.328 |
| **PTT** | 0.914 | 1.094 | **Chronic pulmonary disease** | 0.978 | 1.022 | **Malignant cancer** | 0.952 | 1.051 |
| **LHR** | 0.764 | 1.308 | **WBC** | 0.949 | 1.054 | **Liver disease** | 0.749 | 1.335 |
|  |  |  | **Hematocrit** | 0.854 | 1.171 | **WBC** | 0.947 | 1.056 |
|  |  |  | **Creatinine** | 0.594 | 1.685 | **Creatinine** | 0.554 | 1.804 |
|  |  |  | **BUN** | 0.576 | 1.737 | **BUN** | 0.536 | 1.864 |
|  |  |  | **PTT** | 0.994 | 1.118 | **PTT** | 0.93 | 1.075 |
|  |  |  | **LHR** | 0.765 | 1.307 | **Lactate** | 0.539 | 1.855 |
|  |  |  |  |  |  | **LHR** | 0.581 | 1.721 |
