# Supplementary material for: Lactate to hemoglobin ratio predicts short and long term mortality in critically ill patients with Gastrointestinal bleeding
Source: Sci Rep. 2025 Dec 5;15:43216. doi: 10.1038/s41598-025-27176-6 (PMC12680706; doi:10.1038/s41598-025-27176-6)
Supplement: Supplementary file 4 — Supplementary Material 4 [file 41598_2025_27176_MOESM4_ESM.docx]

| **Outcomes** | **AUC** | **95%CI** | **P** | **Youden index** | **Cutoff** | **Sensitivity** | **Specificity** |
| --- | --- | --- | --- | --- | --- | --- | --- |
| 7d mortality | 0.694 | (0.652,0.737) | ＜0.001 | 0.292 | 0.2057 | 0.754 | 0.538 |
| 28d mortality | 0.657 | (0.626,0.688) | ＜0.001 | 0.224 | 0.1937 | 0.698 | 0.526 |
| 365d mortality | 0.637 | (0.608,0.666) | ＜0.001 | 0.211 | 0.2154 | 0.581 | 0.630 |

**Supplementary material S5 Optimal cut-offs by best Youden index analysis**

**Notes:** AUC: Area under the curve. 95% CI: 95% confidence interval. Youden index: A measure of test accuracy, calculated as sensitivity + specificity – 1. Cutoff: Optimal threshold determined by the Youden index for the lactate-to-hemoglobin ratio (LHR). Sensitivity: Probability of correctly identifying patients with the outcome. Specificity: Probability of correctly identifying patients without the outcome.
