# Supplementary material for: Lactate to hemoglobin ratio predicts short and long term mortality in critically ill patients with Gastrointestinal bleeding
Source: Sci Rep. 2025 Dec 5;15:43216. doi: 10.1038/s41598-025-27176-6 (PMC12680706; doi:10.1038/s41598-025-27176-6)
Supplement: Supplementary file 5 — Supplementary Material 5 [file 41598_2025_27176_MOESM5_ESM.docx]

**Supplementary material S4 AUC with DeLong test**

| **Endpoint** | **AUC1** | **AUC2** | **ΔAUC** | **P** |
| --- | --- | --- | --- | --- |
| **7d mortality** | 0.803 | 0.807 | 0.004 | 0.003 |
| **28d mortality** | 0.794 | 0.796 | 0.002 | 0.018 |
| **365d mortality** | 0.738 | 0.747 | 0.009 | < 0.001 |

**Notes: AUC1:**clinical model + LHR; **AUC2:** clinical model + lactate
